# Microstructure-sensitive critical plastic strain energy density criterion for fatigue life prediction across various loading regimes

**DOI:** 10.1098/rspa.2019.0766

**Published:** 2020-04-01

**Authors:** Ritwik Bandyopadhyay, Veerappan Prithivirajan, Alonso D. Peralta, Michael D. Sangid

**Affiliations:** 1 School of Aeronautics and Astronautics, Purdue University, West Lafayette, IN, USA; 2 Honeywell Aerospace, Phoenix, AZ, USA

**Keywords:** fatigue life, crystal plasticity finite element method, plastic strain energy density, Bayesian inference method, Markov chain Monte Carlo, Metropolis–Hastings algorithm

## Abstract

In the present work, we postulate that a critical value of the stored plastic strain energy density (SPSED) is associated with fatigue failure in metals and is independent of the applied load. Unlike the classical approach of estimating the (homogenized) SPSED as the cumulative area enclosed within the macroscopic stress–strain hysteresis loops, we use crystal plasticity finite element simulations to compute the (local) SPSED at each material point within polycrystalline aggregates of a nickel-based superalloy. A Bayesian inference method is used to calibrate the critical SPSED, which is subsequently used to predict fatigue lives at nine different strain ranges, including strain ratios of 0.05 and −1, using nine statistically equivalent microstructures. For each strain range, the predicted lives from all simulated microstructures follow a lognormal distribution. Moreover, for a given strain ratio, the predicted scatter is seen to be increasing with decreasing strain amplitude; this is indicative of the scatter observed in the fatigue experiments. Finally, the lognormal mean lives at each strain range are in good agreement with the experimental evidence. Since the critical SPSED captures the experimental data with reasonable accuracy across various loading regimes, it is hypothesized to be a material property and sufficient to predict the fatigue life.

## 1. Introduction

Engineering components, subjected to cyclic loading, accumulate damage over the period of their operation owing to the irreversibilities associated with microscopic cyclic plasticity. Such degradation is usually referred to as fatigue and is associated with a finite safe-life operation of the component [1,2]. The estimation of the safe life, referred to as fatigue life throughout this article, is one of the critical tasks in the successful design of components. Especially for aircraft structures, where the factor of safety is almost equal to 1, accurate prediction of fatigue life is crucial.

In the past 100 years, scores of researchers offered numerous criteria, including simple phenomenological relationships and elegant physics-based models, to predict fatigue life at a specified loading condition. Basquin [3] proposed a phenomenological model relating applied stress amplitude to the fatigue life via a power-law relationship. Later, Coffin [4] and Manson [5] emphasized the importance of plastic strain, as opposed to stress, to predict life in the low cycle fatigue regime. They related plastic strain amplitude to fatigue life via a similar power-law relationship which is widely used in the industry to date. In his classic paper on cyclic plasticity, Morrow [6] commented that at the microscopic level neither the plastic strain (arising from the to-and-fro motion of the dislocations) nor the shear stress (resistance to the motion of the dislocations) was individually responsible for fatigue damage accumulation. He postulated that the plastic work was a composite measure of fatigue damage and thus a unified metric for life prediction. Based on the data from a series of fatigue experiments, Morrow proposed another power-law relationship relating plastic work or energy to fatigue life. Following the footsteps of Morrow, a series of researchers employed a similar energy-based approach to predict fatigue life [7–31]. In each of these works, plastic work or energy was computed from the area enclosed within macroscopic stress–strain hysteresis loops.

Fatigue failure can be physically considered as a sequence of three successive events; namely, crack initiation, crack growth/propagation and the final fracture. In the second half of the last century, many research efforts were centred on understanding the physics of crack initiation. Based on extensive research of FCC materials, it was discovered that persistent slip bands (PSBs) were primarily responsible for fatigue crack initiation [32]. In 1956, Thompson *et al*. [33] first described PSBs as specific slip characteristics that would reappear on the surface during cyclic loading even after removing these features by electropolishing. They observed that cracks originated mostly in the interface between the PSB and the matrix. It was also found that the early stage of crack growth was strongly influenced by microstructural attributes (e.g. grain size distribution, texture, etc.) ahead of the crack tip [1,2]. Therefore, microstructural features or characteristics should undoubtedly play a key role in fatigue life prediction. However, in all of the energy-based approaches, as mentioned before, heterogeneities arising in the plastic strain energy at the microstructural length scale were not considered. Instead, a homogenized plastic strain energy density, obtained from the macroscopic stress–strain response, was regarded as a metric for life estimation.

With the advent of high-performance computing, researchers have used crystal plasticity finite element (CPFE) and molecular dynamics (MD) simulations to offer microstructure-sensitive fatigue life prediction frameworks. For example, inspired by the critical plane approach proposed by Fatemi & Socie [34], McDowell and co-workers suggested a fatigue indicative parameter accounting for microstructural heterogeneities in CPFE simulations [35–42]. Sinha & Ghosh [43] proposed a cyclic ratcheting-based model using CPFE simulation results. Further, Ghosh and co-workers used effective traction-based approaches to predict crack initiation during dwell fatigue in titanium alloys [44,45]. On the other hand, using MD simulations, Sangid *et al*. introduced a PSB-driven failure metric for crack initiation [46] and fatigue life prediction [47]. Building on this approach, Yeratapally *et al*. [48] used both MD and CPFE simulations to propose another framework combining two different length scales simultaneously (atomic and mesoscopic) and paying particular attention to the role played by twin boundaries in nickel-based superalloys. All of these models capture the physics of crack initiation and satisfactorily predict life. However, these models either involve physical parameters which are very difficult to measure from currently available experiments or include non-physical parameters which are impossible to calibrate using experiments. Thus, calibration of these models poses an unavoidable hindrance towards their applicability in industry.

Almost four decades after Morrow's classic paper on cyclic plasticity [6], researchers have returned to energy-based approaches to address microstructure-sensitive fatigue life performance. For example, building on the work by Korsunsky *et al*. [49], Wan *et al*. [50] proposed a Griffith- or Stroh-type critical stored energy density (energy per unit area) as a mesoscale driving force for crack initiation and fatigue life prediction in 2014. Using the same model, Jiang *et al*. [51] and Chen *et al*. [52] predicted multiple crack initiations around non-metallic inclusions within nickel-based superalloys, and Wilson *et al*. [53–55] predicted crack growth within polycrystalline microstructures. In a separate work, Cruzado *et al*. [56] proposed a similar model involving two material parameters. The microstructure-sensitive energy-based approach, introduced by Dunne and co-workers [49–55], is undoubtedly promising because it (i) is inherently physics based, (ii) involves only one parameter, namely critical stored energy density, (iii) takes care of microstructural heterogeneities, and (iv) offers a path forward for calibration via advanced experimental techniques. However, several aspects associated with such an approach have not been addressed so far in the literature. First, it has not been investigated whether a single critical stored energy density value can reasonably predict fatigue lives at multiple loading regimes, including low and high cycle fatigue and different stress/strain ratios. Second, experimental fatigue life data at a given stress/strain amplitude show a scatter which is typically observed to be (i) lognormally distributed [57] and (ii) increasing with decreasing applied stress/strain amplitude. A microstructure-sensitive framework should be able to capture both trends in the scatter; however, this has not been verified systematically. The primary focus of the current work is to address these two unexplored aspects.

The amount of work done by the externally applied forces during cyclic loading can be equated to the summation of the elastically stored energy, which is recoverable upon unloading, and the internal plastic work, which is non-recoverable upon unloading. The material stores a portion of the internal plastic work by forming dislocation structures and sub-structures, such as PSBs, and the rest is primarily dissipated as heat energy. In the context of fatigue failure, it is the stored portion of the internal plastic work that is of interest. Here, it is postulated that there exists a critical value of the stored plastic strain energy density (SPSED) for a material, which is associated with fatigue failure and is independent of applied loading. In the present work, based on CPFE simulations, the critical plastic strain energy density is calibrated using experimentally available fatigue life data for 718Plus, a nickel-based superalloy. Subsequently, the calibrated energy is used to predict fatigue lives at several loading regimes, including multiple applied strain amplitudes and strain ratios. Moreover, statistically equivalent microstructures (SEMs) are used to generate a scatter in the prediction to test for (i) lognormal distribution at a given applied strain and (ii) increasing spread in the prediction with decreasing applied strain. With this, the paper is structured as follows. The CPFE simulation framework including the materials processing information, the generation of SEMs and the description of the crystal plasticity model is detailed in §2. In §3, the calculation of the plastic strain energy density and the identification of the critical location for failure are discussed. A Bayesian calibration framework is outlined in §4 to estimate the critical plastic strain energy density and quantify the associated uncertainties. In §5, the results for the critical plastic strain energy density and predicted fatigue lives are reported. In §6, the results are discussed. Finally, concluding remarks are presented.

## 2. Crystal plasticity finite element simulation framework

In the present section, material processing information for 718Plus is summarized. Next, the steps associated with the generation of discretized polycrystalline microstructures are outlined. Subsequently, the kinematic and kinetic equations associated with the crystal plasticity model are presented. Finally, a slip system-based averaging scheme is discussed to regularize the numerical results from the CPFE simulations.

### (a) Materials processing

**Figure 1.**
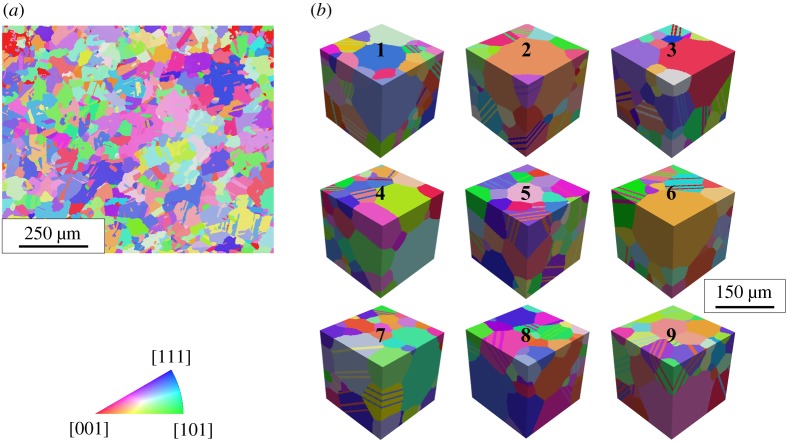
(*a*) EBSD characterization of the nickel-based superalloy 718Plus. (*b*) SEMs corresponding to the EBSD map. (Online version in colour.)

In this study, the material under consideration is 718Plus, a nickel-based superalloy. In particular, this material is processed via additive manufacturing, specifically selective laser melting, using an EOS M280 laser powder-bed machine at Honeywell Aerospace, Phoenix, AZ, USA. The build parameters are optimized based on process modelling, discussed in [58], to result in minimal porosity [59]. The optimized parameter values are 300 W laser power, 0.09 mm hatch spacing, 1.65 m s^−1^ laser speed and stripe width of 5 mm. The powder used is ATI 718Plus® [60], which is referred to as 718Plus throughout this paper. Following the build, the material is subjected to a stress relief heat treatment at 1010°C for 1.5 h with a flue gas quench followed by a hot isostatic pressure cycle at 1163°C and 103 MPa for 4 h. Subsequently, the material is solution heat treated at 1010°C for 2 h with a flue gas quench and aged at 788°C for 8 h followed by a ramp down at 39°C h^−1^ to achieve a temperature of 704°C. At 704°C, the material is held for 8 h then cooled in a furnace. This heat treatment procedure has been shown to provide optimal microstructures and preferred strength characteristics [59]. The resulting microstructure, obtained via electron backscatter diffraction (EBSD), is shown in figure 1*a*.

The bulk material is machined into specimens with a gauge length and diameter of 12.7 mm and 5 mm, respectively. Each specimen is polished to mitigate surface defects due to roughness. A total of 76 samples are tested according to ASTM E606 standards [61]. The fatigue testing is performed on smooth bars at room temperature with a constant amplitude loading frequency of 0.33 Hz. The load values are recorded and, from this information, crack initiation and failure are defined at 10% and 50% load drops, respectively, in the stabilized maximum stress values. Experiments are performed at applied strain ratios of 0.05 and −1 across a range of applied strain amplitudes necessary to achieve a complete strain–life curve, as shown in §5b. From the fractography analyses, the failure locations were typically associated with grain facets; hence, crack initiation was not observed at features such as pores or inclusions. Therefore, microstructural defects, such as pores and inclusions, are not modelled in the CPFE analysis.

### (b) Generation of discretized polycrystalline microstructures

The generation of the discretized polycrystalline microstructures is a two-step process. First, a virtual three-dimensional polycrystalline microstructure is created. After that, the microstructure is discretized using a finite element meshing software.

The creation of a virtual three-dimensional polycrystalline microstructure is a multi-step iterative process [62]. First, microstructural attributes (grain size distribution, twin area fraction, etc.) are obtained from the EBSD characterization of the material. Second, DREAM.3D software is used to create a synthetic volume with slightly higher grain size distribution [63]. Third, twins are inserted along one of the randomly chosen {111} planes in randomly chosen parent grains within the synthetic volume [64]. The orientation of the inserted twin is assigned to ensure 60° misorientation between the twin and the parent grain. The grain size distribution within the synthetic volume changes after the insertion of the twins. Therefore, the second, third and fourth steps are repeated iteratively until the final grain size distribution and twin area fraction from the virtual three-dimensional polycrystalline microstructure match the same obtained from the EBSD characterization. For the present work, nine virtual three-dimensional polycrystalline microstructures are synthesized (figure 1*b*). These are cubes of dimension 180 µm and are statistically equivalent to the microstructure features characterized in the EBSD map. Hence, these virtual three-dimensional polycrystalline microstructures are called SEMs representing the strength properties of the material and referred to as SEMs throughout this paper. Microstructural statistics of the SEMs are summarized in table 1.

**Table 1.** Microstructural statistics of the SEMs.

			grain size (µm)		
SEM	number of grains	minimum	mean	maximum	twin area fraction
1	135	15.67	35.13	134.19	0.50
2	139	17.68	34.59	159.08	0.51
3	139	14.24	35.76	132.30	0.51
4	140	16.76	34.65	138.22	0.49
5	140	19.92	36.14	141.83	0.53
6	141	16.48	35.58	137.86	0.54
7	137	16.37	35.40	140.29	0.54
8	146	16.18	35.43	136.82	0.51
9	133	16.67	35.30	136.56	0.55

**Figure 2.**
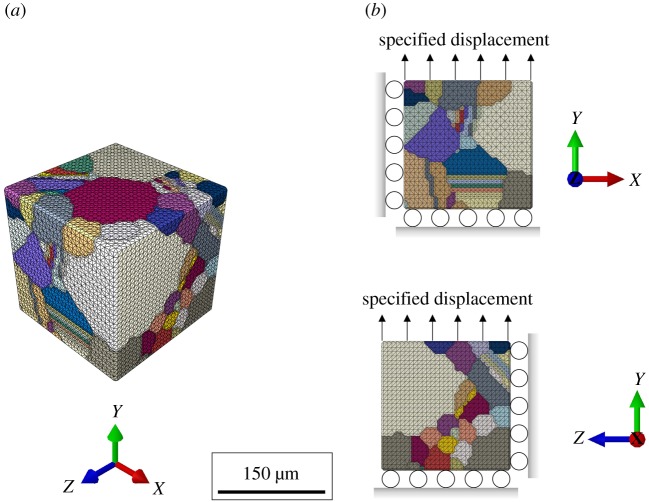
(*a*) Discretized SEM. (*b*) Applied boundary conditions on the discretized polycrystalline microstructure model. (Online version in colour.)

After performing Laplacian smoothing of the grain boundaries, the surface mesh for each SEM is exported from DREAM.3D. Subsequently, the surface mesh is used to perform volume meshing of the SEMs using Gmsh software [65]. For the present work, computationally efficient linear tetrahedron elements (C3D4) are chosen, and the mean element size is found to be approximately 3.75 µm. The choice of the C3D4 elements often leads to numerical artefacts in the results from an elasto-plastic analysis. A possible mitigation strategy, adopted in the present work, is discussed in §2d. A discretized version of SEM 1 is shown in figure 2*a*.

### (c) Crystal plasticity model and parameter estimation

The total deformation gradient (**F**) associated with the elasto-plastic deformation of a solid body can be decomposed as [66]

$$F=F^{e}\cdot F^{p}.$$

Here, $F^{p}$ and $F^{e}$ are deformation gradients associated with the plastic and elastic parts, respectively. Further, the plastic velocity gradient $\left(L^{p}\right)$ is related to $F^{p}$ as

$$L^{p}={\dot{F}}^{p}\cdot {\left(F^{p}\right)}^{-1}.$$

Within a crystal plasticity framework, the plastic velocity gradient $\left(L^{p}\right)$ is written as a sum of the contributions from all active slip systems [67]. Since 718Plus is an FCC material, the sum is performed over all ⟨110⟩{111} slip systems as follows:

2.3 $$L^{p}=\overset{12}{\underset{k=1}{\sum }}{\dot{\gamma }}^{k}s^{k}⊗n^{k}.$$

Here, ${\dot{\gamma }}^{k}$, $s^{k}$ and $n^{k}$ are the shearing rate, the unit vector along the slip direction and the unit vector along the slip plane normal associated with the *k*th slip system. The shearing rate $\left({\dot{\gamma }}^{k}\right)$ for a slip system is related to the resolved shear stress $\left(\tau^{k}\right)$ on the same slip system via a flow rule. In the present work, a Hutchinson-type flow rule [68] is used of the following form:

2.4 $${\dot{\gamma }}^{k}={\dot{\gamma }}_{0}{\left|\frac{\tau^{k}-\chi^{k}}{g^{k}}\right|}^{n}\text{sgn}(\tau^{k}-\chi^{k}).$$

Here, ${\dot{\gamma }}_{0}$ is a reference shearing rate; $g^{k}$ and $\chi^{k}$ are the reference and back stresses associated with the *k*th slip system, respectively; and *n* is the inverse strain rate sensitivity exponent. Both reference and back stresses evolve with plastic deformation. In the present work, the evolutions are modelled with an Armstrong–Fredrick-type equation [69,70] and are given by

2.5 $${\dot{g}}^{k}=H\overset{12}{\underset{m=1}{\sum }}q^{km}|{\dot{\gamma }}^{m}|-H_{d}g^{k}\overset{12}{\underset{m=1}{\sum }}|{\dot{\gamma }}^{m}|$$

and

2.6 $${\dot{\chi }}^{k}=A{\dot{\gamma }}^{k}-A_{d}\chi^{k}|{\dot{\gamma }}^{k}|.$$

**Figure 3.**
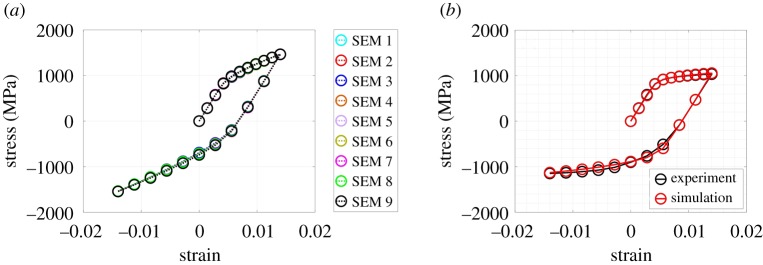
(*a*) Macroscopic stress–strain response of all SEMs using the crystal plasticity parameters reported in [72]. (*b*) Experimental and simulated macroscopic stress–strain response from SEM 1 using calibrated crystal plasticity parameters for 718Plus in this study. (Online version in colour.)

Here, *H* and *A* are the direct hardening coefficients for the reference and back stresses, respectively; *H_d_* and *A_d_* are the dynamic recovery coefficients for the reference and back stresses, respectively. Further, $q^{km}$ is the hardening matrix, where diagonal terms represent self-hardening and off-diagonal terms are associated with latent hardening ($q^{km}=1$ if *k *= *m*, otherwise $q^{km}=1.2$ [71]). All the evolution equations, as described above, are discretized and implemented within an ABAQUS user-defined material subroutine (UMAT). For simplicity, the initial values of the reference and back stresses are assumed to be the same for all slip systems. With this, there are eight crystal plasticity parameters (${\dot{\gamma }}_{0}$, *n*, g(0), *H*, $H_{d}$, χ(0), *A*, *A_d_*) which are to be calibrated. Starting with the crystal plasticity parameters reported for additively manufactured IN718 in [72], a Matlab-based genetic algorithm framework, as described in [62], is used to identify an optimal set of these parameters such that the homogenized macroscopic response of the model matches the experimentally obtained macroscopic stress–strain response of the material. During simulations, normal displacements on three mutually orthogonal adjacent surfaces of the SEMs are restricted, and normal displacement is specified on another face, as shown in figure 2*b*, to mimic the uniaxial applied loading condition. The anisotropic elastic constants are adopted from [73]. For the crystal plasticity parameters reported in [72], the macroscopic responses of all SEMs are similar (figure 3*a*). Therefore, only SEM 1 is used to calibrate the crystal plasticity parameters. The anisotropic elastic constants along with the calibrated crystal plasticity parameters are reported in table 2. The simulated macroscopic stress–strain response with the calibrated crystal plasticity parameters is compared with the experimental stress–strain data for 718Plus in figure 3*b*. A good agreement between the simulated and experimental stress–strain response is observed in figure 3*b*. Uncertainty quantification for each parameter of the present crystal plasticity model is discussed in detail by the authors in [62].

**Table 2.** Anisotropic elastic constants and calibrated crystal plasticity parameters.

parameter	value	parameter	value
*C*_11_	239.57 GPa	*H*	1885 MPa
*C*_12_	141.18 GPa	*H_d_*	8
*C*_44_	107.04 GPa	*A*	5997 MPa
${\dot{\gamma }}_{0}$	4.25 × 10^−4^ s^−1^	*A_d_*	31
*n*	12	*χ*(0)	10.2 MPa
*g*(0)	290.3 MPa	—	—

### (d) Slip system averaging scheme

**Figure 4.**
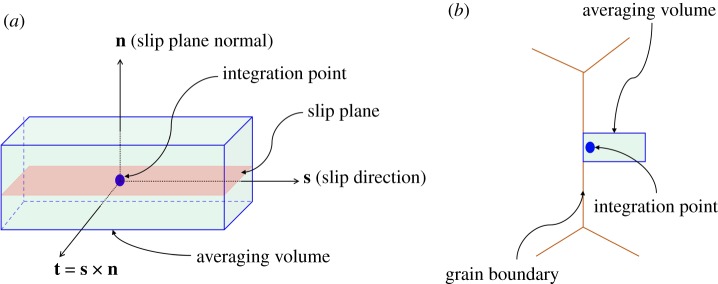
(*a*) Slip system-based averaging scheme at an arbitrary integration point. (*b*) The averaging volume for an integration point near the grain boundary is not allowed to cross the grain boundary. (Online version in colour.)

The mechanical field variables (e.g. stress, plastic strain accumulation) from CPFE simulations often show spurious numerical oscillations within the simulation domain [72,74]. Such behaviour can be related to one or a combination of the following: (i) volumetric locking or shear locking owing to the choice of the linear tetrahedron (C3D4) elements [74], (ii) lack of mesh refinement near the grain boundaries across which high gradients in field variables are expected, and (iii) the effect of the boundary conditions on the elements near the corresponding surfaces. Under such a situation, one needs to adopt a homogenization scheme that can regularize numerical oscillations, preserving the inherent spatial gradient associated with the field variable. One possibility is a slip system-based averaging [72,75–79]. To illustrate the averaging scheme, let us consider an integration point as shown in figure 4*a*. Three directions are identified passing through the integration point; namely, slip direction (**s**), slip plane normal (**n**) and a transverse vector (**t**) on the slip plane such that these directions form a right-hand coordinate system at the integration point. Now, a small rectangular cuboid is considered, with the centroid coinciding with the integration point and the faces parallel to the three directions defined before. The region enclosed by the rectangular cuboid is called the averaging volume. The field variable is averaged over all the elements whose centroids lie within the averaging volume. One can construct 12 such volumes, one for each slip system, around the same integration point. After performing averaging for all 12 volumes, the maximum (by magnitude) of the averaged values is assigned as the slip system-averaged number to that integration point. If an integration point is near the grain boundary, the averaging volume is not allowed to cross the grain boundary (figure 4*b*) to preserve the gradient, which is often expected across the grain boundary. The size of the averaging volume is dependent on the size of the finite elements. Presently, the lengths of the averaging volume are defined by 4, 3, 3 elements along the slip direction (**s**), slip plane normal (**n**) and transverse direction (**t**), respectively. Such an averaging scheme is implemented for all relevant field variables under present consideration during the post-processing phase of the CPFE fatigue simulations.

## 3. Critical plastic strain energy density criterion for fatigue life estimation

In crystal plasticity analysis, the plastic strain energy density at a material point **x** is defined as a sum of contributions from all slip systems and its increment over the *y*th cycle is written as

3.1 $$\Delta w_{y}^{p}(x)=\overset{\;}{\underset{y\text{th cycle}}{\oint }}\left(\overset{12}{\underset{k=1}{\sum }}|\tau^{k}(x,t)\;{\dot{\gamma }}^{k}(x,t)|\right)\text{d}t.$$

Here, $\Delta w_{y}^{p}(x)$ is the increment of the plastic strain energy density at the point **x** over the *y*th loading cycle. Now, the total accumulated plastic strain energy density after the *y*th cycle at the same point, $w_{y}^{p}(x)$, is expressed as

3.2 $$w_{y}^{p}(x)=\;\overset{y}{\underset{i=1}{\sum }}\Delta w_{i}^{p}(x).$$

In the present work, it is postulated that, if the material fails due to fatigue at $x=x^{*}$ after *N_f_* cycles, $w_{N_{f}}^{p}(x^{*})$ is a material property and is independent of the applied loading conditions. Further, $w_{N_{f}}^{p}(x^{*})$ is referred to as the critical plastic strain energy density and is denoted as $W_{\text{critical}}^{p}$. With experimentally known fatigue life, one needs to first calibrate $W_{\text{critical}}^{p}$. Thereafter, the calibrated critical plastic strain energy density can be used to predict the fatigue life at several loading conditions.

It is practically impossible to run CPFE simulations for thousands of cycles to track the evolutions of $w_{i}^{p}(x)$ and $\Delta w_{i}^{p}(x)$ with the number of cycles. However, it is well known from experiments that the hysteresis behaviour saturates upon cyclic loading, when stable dislocation structures and sub-structures are formed within the material [1]. Here, *N_s_* is defined as the number of cycles required for saturation of the hysteresis loops. From CPFE simulations, *N_s_* is identified based on saturation of $\Delta w_{i}^{p}(x)$. Subsequently, all simulations are continued until *N_s_* cycles to obtain $w_{N_{s}}^{p}(x)$ and $\Delta w_{N_{s}}^{p}(x)$. Since hysteresis loops saturate relatively quickly, material points experience an almost steady increment of plastic strain energy density for each loading cycle over a significantly large portion of the material's life. Therefore, from CPFE simulations, failure location, $x^{*}$, is identified by the location of the maximum value^1^ of $\Delta w_{N_{s}}^{p}(x)$. Finally, $w_{N_{s}}^{p}(x^{*})$ is extrapolated to the experimentally known fatigue life, $N_{f}^{\text{expt}}$, using the following linear relationship to obtain $w_{N_{f}}^{p}(x^{*})$ from CPFE simulations as

3.3 $$w_{N_{f}}^{p}(x^{*})=w_{N_{s}}^{p}(x^{*})+(N_{f}^{\text{expt}}-N_{s})\;\Delta w_{N_{s}}^{p}(x^{*}).$$

Subsequently, $w_{N_{f}}^{p}(x^{*})$, as obtained from equation (3.3), is used to calibrate $W_{\text{critical}}^{p}$ for 718Plus using a Bayesian inference method as described in the next section.

## 4. Bayesian calibration of the critical plastic strain energy density

Variability is inherent in the experimental fatigue life data of a metallic material for a given loading condition. Such scatter in fatigue life data primarily comes from the variability in the statistical strength within the material associated with the local microstructure (e.g. grain size distributions, texture, pores, inclusions, precipitates) paired with fatigue crack initiation as a locally driven phenomenon. Therefore, in the end one has multiple $W_{\text{critical}}^{p}$ values for the same material if calibration is performed by taking all experimental fatigue life data and simulated $w_{N_{s}}^{p}(x^{*})$, $\Delta w_{N_{s}}^{p}(x^{*})$ information from all SEMs. Since $W_{\text{critical}}^{p}$ is hypothesized to be a material property, just like any other material property, one may expect a distribution of the same as opposed to a constant value. Hence, one of the objectives of the present work is to determine a distribution of the $W_{\text{critical}}^{p}$ using a suitable method. Subsequently, the statistically expected value of $W_{\text{critical}}^{p}$ can be used as a representative number for the material property.

The Bayesian inference method is a powerful tool to calibrate model parameters when variability exists in the input and scatter is inevitable in the prior experimental knowledge of the output [80]. In the present work, the fatigue life prediction model is given by

4.1 $$N_{f}^{\text{predict}}(\beta ,W_{\text{critical}}^{p})=\frac{W_{\text{critical}}^{p}-w_{N_{s}}^{p}(\beta ,x^{*})}{\Delta w_{N_{s}}^{p}(\beta ,x^{*})}+N_{s}(\beta ).$$

Here, β is a set of parameters which define the loading conditions, such as the applied strain range, strain ratio, temperature; $w_{N_{s}}^{p}(\beta ,x^{*})$, $\Delta w_{N_{s}}^{p}(\beta ,x^{*})$ and $N_{s}(\beta )$ come from the CPFE simulations; and $W_{\text{critical}}^{p}$ is the *only* model parameter which is to be calibrated. For a given loading situation, i.e. β, variability is expected in $w_{N_{s}}^{p}(\beta ,x^{*})$ and $\Delta w_{N_{s}}^{p}(\beta ,x^{*})$ from different SEMs and scatter is experimentally observed in $N_{f}^{\text{expt}}(\beta )$. Therefore, the Bayesian inference method is an appropriate tool to calibrate $W_{\text{critical}}^{p}$.

From traditional fatigue experiments, it is well known that scatter in fatigue life is dependent on the applied strain range showing an increasing trend with decreasing applied strain range [57]. Therefore, it is more appropriate to use a subset of the experimental fatigue life dataset at a higher applied strain range to calibrate $W_{\text{critical}}^{p}$ to result in less uncertainty. For the present work, the fatigue experiments are carried out from 0.3% to 1.2% applied strain range. Therefore, only the 1.2% applied strain range dataset is used for calibration, and the rest of the data are kept *untouched* for subsequent verification.

**Figure 5.**
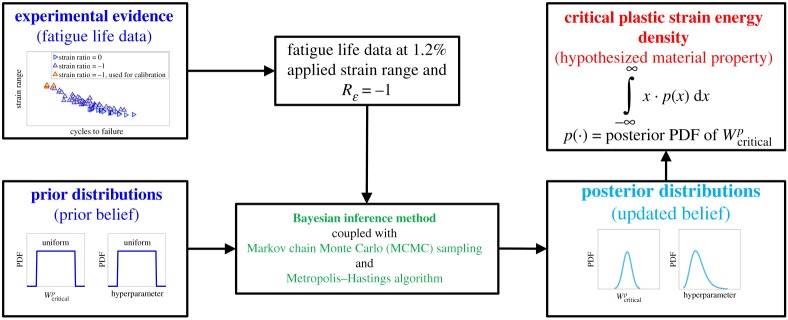
A schematic of the Bayesian inference framework to calibrate the critical plastic strain energy density. (Online version in colour.)

A broad overview of the Bayesian calibration method is shown in figure 5. Key inputs to the calibration framework are experimental knowledge (i.e. fatigue life data) and prior distribution of the model parameter $\left(W_{\text{critical}}^{p}\right)$ based on experience or prior belief. The output from the calibration framework is the posterior distribution, which is the calibrated distribution of the model parameter. Finally, the expected value of the model parameter $\left(W_{\text{critical}}^{p}\right)$ is obtained based on its posterior distribution.

The experimentally observed fatigue life $\left(N_{f}^{\text{expt}}\right)$ and prediction from the model $\left(N_{f}^{\text{predict}}\right)$ can be related using the following equation:

4.2 $$N_{f}^{\text{expt}}(\beta )=N_{f}^{\text{predict}}(\beta ,W_{\text{critical}}^{p})+\delta (\beta )+e(\beta ).$$

Here, δ(β) accounts for the model's inability to capture the correct physics, including limitations associated with the phenomenological evolution equations of the crystal plasticity model; e(β) is the error associated with the experimental measurements and uncertainty. The model discrepancy, δ(β), can be assumed to be independently and identically distributed, and hence is considered as normally distributed with zero mean and standard deviation, *s*_1_ (unknown). Similarly, without loss of generality, e(β) can be assumed to be independently and identically distributed, and hence is considered as normally distributed with zero mean and standard deviation, *s*_2_ (unknown). Since the summation of two normal distributions with identical mean is also a normal distribution, the model discrepancy and experimental error terms are combined to form another normal distribution with zero mean and standard deviation, *s* (unknown). Consequently, in addition to $W_{\text{critical}}^{p},$ the parameter *s* is also required to be calibrated. Therefore, the new augmented parameter set is given by $\alpha =\{W_{\text{critical}}^{p},\;s\}$. The additional parameter, *s*, is also known as a hyper-parameter. It is clarified that the fatigue life prediction model, given by equation (4.1), still involves only *one* parameter. However, because of the choice of the current calibration framework, the model is required to be calibrated by considering equation (4.2), which relates stochastic aspects of the model predictions and experimental observations. Consequently, an additional unknown standard deviation parameter, *s*, is required to be calibrated. After calibration, *s* has no role to play in the subsequent fatigue life prediction.

In the present work, the Bayesian calibration framework is composed of three ingredients, namely the Bayesian inference method, Markov chain Monte Carlo (MCMC) sampling and the Metropolis–Hastings algorithm. A brief description of each of these ingredients and their roles in the Bayesian calibration framework are provided below.

### (a) Bayesian inference method

The Bayesian inference technique updates the prior distribution of the parameter set α based on the experimental evidence and likelihood function. The posterior distribution is obtained as output using

4.3 $$\pi (\alpha \text{|}D)=\frac{\pi (D|\alpha )\pi_{0}(\alpha )}{\pi (D)}=\frac{\pi (D\text{|}\alpha )\pi_{0}(\alpha )}{\int \pi (D\text{|}\alpha )\pi_{0}(\alpha )\;\text{d}\alpha }.$$

Here, $\pi_{0}(\alpha )$ represents the prior distribution of the parameter set α and π(D|α) represents the likelihood of observing experimental data, *D*, for the given parameter set α; and π(α|D) is the posterior distribution of the parameter set α based on experimental evidence *D* and likelihood term π(D|α). Since the error terms, as defined in equation (4.2), are assumed to be independently and identically distributed, the likelihood term is expressed as [80]

4.4 $$\pi (D\text{|}\alpha )=\frac{1}{{\left(2\pi s^{2}\right)}^{q/2}}\exp\left(-\frac{\text{SSE}(\alpha )}{2s^{2}}\right).$$

Here, *s* is the hyper-parameter as defined before; SSE(α) is the sum of squares of errors for the parameter set α and is expressed as

4.5 $$\text{SSE}(\alpha )=\overset{m}{\underset{i=\;1}{\sum }}\overset{k}{\underset{j\;=\;1}{\sum }}{\left(N_{i}^{\text{expt}}-N_{j}^{\text{predict}}(\alpha )\right)}^{2}.$$

In equation (4.5), $N_{i}^{\text{expt}}$ is the *i*th experimental fatigue life in a set of *m* data and $N_{j}^{\text{predict}}$ is the predicted life corresponding to the *j*th SEM in a set of *k*(=9) SEMs at a given loading situation. The parameter *q* in equation (4.4) is related to *m* and *k* as

4.6 q=m⋅k.

### (b) Markov chain Monte Carlo sampling and Metropolis–Hastings algorithm

For many practical cases, it is challenging to evaluate the integral in the denominator of equation (4.3) analytically or using numerical quadrature. In such situations, the MCMC sampling method is preferred to obtain posterior distributions for the parameter set α. A Markov chain is a sequence of random variables that satisfies the Markov property that the *k*th term in the sequence depends only on the (k−1)th term. The *k*th term is proposed via the Monte Carlo sampling method, which is based on a proposal distribution. The decision whether the new proposed candidate point will be accepted as the *k*th term in the sequence is taken based on the Metropolis–Hastings algorithm [81,82]. Stepwise computer implementation of the algorithm has been discussed by Yeratapally *et al*. [83] in relation to the uncertainty quantification of a microstructure-sensitive fatigue life prediction model. However, for the sake of completeness and continuity, important aspects and equations of the Metropolis–Hastings algorithm are outlined below.

Let, $\alpha_{j}^{k-1}$ be the *j*th parameter (*j* = 1, 2) in the (k−1)th term of the Markov chain and let $\alpha_{j}^{*}$ be the proposed *j*th parameter for the *k*th term in the sequence. Now, the following number is computed:

4.7 $$p=\min\left(1,\frac{\pi (D|\alpha_{j}^{*})\pi_{0}(\alpha_{j}^{*})\pi (\alpha_{j}^{*}|\alpha_{j}^{k-1})}{\pi (D|\alpha_{j}^{k-1})\pi_{0}(\alpha_{j}^{k-1})\pi (\alpha_{j}^{k-1}|\alpha_{j}^{*})}\right).$$

Here, $\pi (\alpha_{j}^{*}|\alpha_{j}^{k-1})$ represents the proposal distribution used to generate the proposed candidate point $\alpha_{j}^{*}$ based on the current candidate point $\alpha_{j}^{k-1}$. Here, the proposal distribution is assumed to be symmetric, for which

4.8 $$\pi (\alpha_{j}^{*}|\alpha_{j}^{k-1})=\pi (\alpha_{j}^{k-1}|\alpha_{j}^{*}).$$

Therefore, equation (4.7) can be simplified and rewritten as follows:

4.9 $$p=\min\left(1,\frac{\pi (D|\alpha_{j}^{*})\pi_{0}(\alpha_{j}^{*})}{\pi (D|\alpha_{j}^{k-1})\pi_{0}(\alpha_{j}^{k-1})}\right).$$

The assumption of a symmetric proposal distribution is a matter of convenience and simplicity. If the posterior distribution is expected to be symmetric, such a choice leads to quick convergence (as discussed later). Otherwise, it may take a longer time to obtain a converged posterior distribution. After obtaining *p* from equation (4.9), a uniformly distributed random number *u* is generated within the interval 0≤u≤1. If u≤p, the proposed candidate is selected as the *k*th term in the sequence, i.e. $\alpha_{j}^{k}=\alpha_{j}^{*}$. Otherwise, the (k−1)th term is assigned as the *k*th term, i.e. $\alpha_{j}^{k}=\alpha_{j}^{k-1}$. This process is continued until convergence is achieved. To check for convergence, *M* Markov chains are run in parallel. If $B_{\alpha_{j}}$ and $W_{\alpha_{j}}$ are the variance of the parameter $\alpha_{j}$ between and within chains, respectively, then an estimate of the variance of the parameter $\alpha_{j}$, $V_{\alpha_{j}}$, is given by

4.10 $$V_{\alpha_{j}}=\frac{M-1}{M}W_{\alpha_{j}}+\frac{1}{M}B_{\alpha_{j}}.$$

Using $V_{\alpha_{j}}$ and $W_{\alpha_{j}}$ a convergence parameter $R_{\alpha_{j}}$ is defined as

4.11 $$R_{\alpha_{j}}=\sqrt{\frac{V_{\alpha_{j}}}{W_{\alpha_{j}}}}.$$

With the increase in the number of Monte Carlo sampling, *k*, in each Markov chain, the parameter $R_{\alpha_{j}}$ approaches unity, resulting in a converged/stationary distribution. Such a converged/stationary distribution is the posterior distribution. The reader is referred to Cross *et al*. [84] for the expressions of $W_{\alpha_{j}}$ and $B_{\alpha_{j}}$.

## 5. Results

### (a) Critical plastic strain energy density

**Figure 6.**
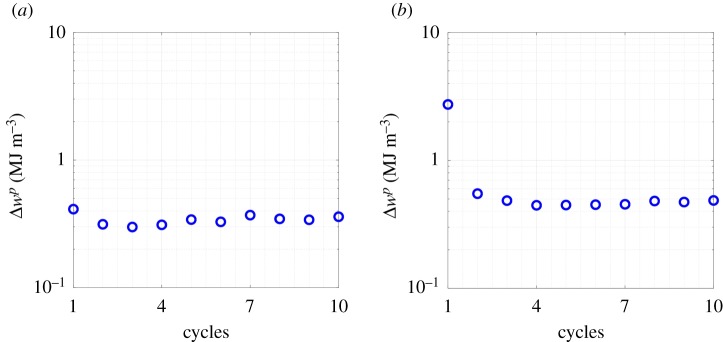
Evolution of plastic strain energy density per cycle with the number of cycles at the critical location within SEM 1 under macroscopically applied strain, Δε=0.5% at (*a*) $R_{\varepsilon }=-1$ and (*b*) $R_{\varepsilon }=0.05$. (Online version in colour.)

**Figure 7.**
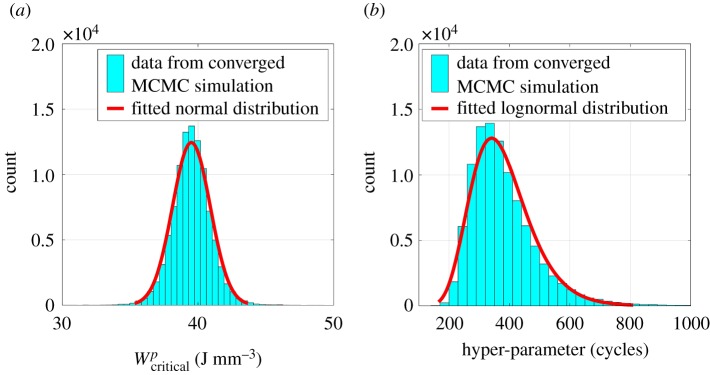
Posterior distributions for (*a*) the critical plastic strain energy density, $W_{\text{critical}}^{p}$, and (*b*) the hyper-parameter, *s*. (Online version in colour.)

For a representative example, evolutions of $\Delta w^{p}$ with the number of cycles at the critical location within SEM 1 under macroscopically applied strain Δε=0.5% at strain ratios $R_{\varepsilon }=-1$ and 0.05 are shown in figure 6. It is evident from figure 6 that $\Delta w^{p}$ saturates immediately after the first cycle. However, simulations are continued until 10 cycles in the present work, resulting in $N_{s}=10$. For Bayesian calibration, the CPFE simulations are performed on all SEMs at Δε=1.2% with $R_{\varepsilon }=-1$ to obtain $w_{10}^{p}(x^{*})$ and $\Delta w_{10}^{p}(x^{*})$. Uniform distributions are assumed as prior distributions for both parameters, and the MCMC simulations are continued until 10^5^ iterations to ensure convergence. Here, convergence is accepted if $R_{\alpha_{j}}<1.005$. Converged posterior distributions are shown in figure 7. From figure 7, it is observed that the posterior data for $W_{\text{critical}}^{p}$ closely follow a normal distribution, whereas the posterior distribution for the hyper-parameter is found to follow a lognormal distribution. Characteristics of the posterior distributions are reported in table 3.

**Table 3.** Prior and posterior distributions for the critical plastic strain energy density and hyper-parameter.

			posterior distribution		
parameter	prior distribution	type	mean	standard deviation	$R_{\alpha_{j}}$
$W_{\text{critical}}^{p}$	uniform, U(0,∞)	normal	39.5 J mm^−3^	1.4 J mm^−3^	1.004
*s*	uniform, U(0,∞)	lognormal	379.0 cycles	104.0 cycles	1.002

### (b) Fatigue life prediction

**Figure 8.**
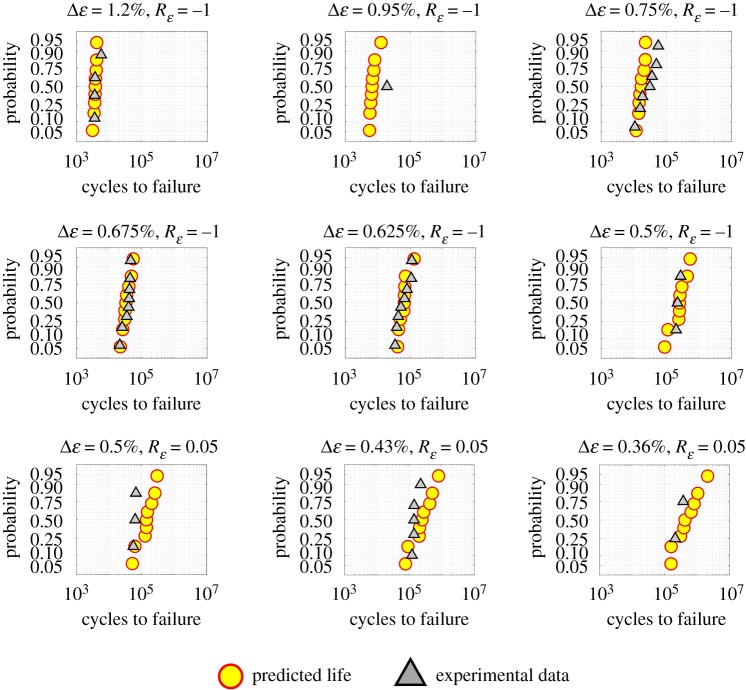
The probability of failure plots considering a lognormal distribution as the reference. (Online version in colour.)

**Figure 9.**
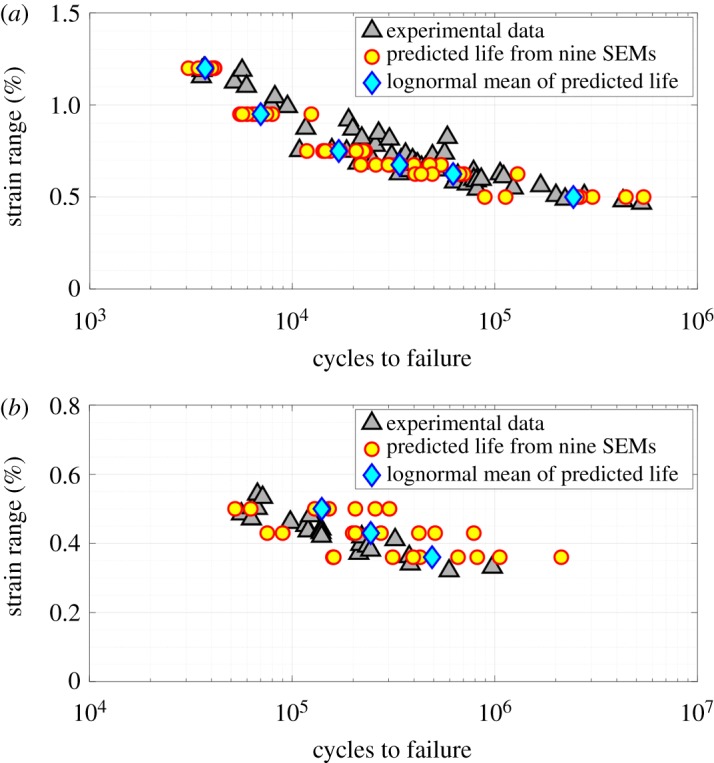
Experimental and predicted fatigue life data at (*a*) $R_{\varepsilon }=-1$ and (*b*) $R_{\varepsilon }=0.05$. (Online version in colour.)

The CPFE simulations are performed for Δε=0.95%, 0.75%, 0.675%, 0.625%, 0.5% at $R_{\varepsilon }=-1$ and for Δε=0.5%, 0.43%, 0.36% at $R_{\varepsilon }=0.05$. From all simulations and all SEMs, $w_{10}^{p}(x^{*})$ and $\Delta w_{10}^{p}(x^{*})$ are obtained. Finally, these values are used within equation (4.1) along with the $W_{\text{critical}}^{p}$ value, as reported in table 3, to predict fatigue lives at each strain range from all SEMs. The probability of failure plots is obtained using the probplot function in Matlab by considering the lognormal distribution as a reference, as shown in figure 8. In figure 8, the vertical axes are depicted in such a way that, if data points are sampled from lognormal distributions, they will be aligned along a straight line. As expected, experimental data points in each plot appear to be aligned along a straight line. Interestingly, all the predicted data points also appear to be aligned along a straight line. Therefore, it can be said that the microstructure-sensitive critical plastic strain energy density criterion consistently predicts lognormally distributed fatigue life across several loading regimes. In each case, predicted lives from all SEMs are fitted to a lognormal distribution. Experimental data, predictions from all SEMs and the lognormal mean of predictions are shown in figure 9. A good agreement between the experimental and predicted lives is seen in figures 8 and 9. Further, an increasing spread in the fatigue life predictions, from an identical set of SEMs, is observed with a decreasing applied strain.

## 6. Discussion

The fatigue life prediction framework, presented in this work, inherently captures the relevant physics or mechanism of the plastic deformation via crystal plasticity constitutive equations. Hence, it can capture the irreversibilities associated with the deformation even if the material is loaded elastically in a macroscopic sense. In other words, the framework can be used for both high cycle fatigue, dominated by microplasticity, and low cycle fatigue, dominated by a large amount of plastic deformation, situations. In this work, dislocation glide is the primary deformation mechanism which has been incorporated in the crystal plasticity framework via equation (2.3). In other material systems, additional deformation mechanisms, such as deformation twinning, might be dominant as well. In those situations, kinematic equations (equations (2.1)–(2.3)) for the crystal plasticity model should be updated appropriately before using equation (3.1). Further, the present approach can, in principle, be extended to situations where the environment plays an important role (e.g. hydrogen embrittlement, corrosion) in the fatigue performance of the material via incorporating suitable constitutive relations in the crystal plasticity model.

The reader is reminded that the CPFE simulation results, i.e. $w_{10}^{p}(x^{*})$ and $\Delta w_{10}^{p}(x^{*})$, are key inputs to the fatigue life prediction model given by equation (4.1). Therefore, the magnitude of the calibrated $W_{\text{critical}}^{p}$ strongly depends on the size of the slip system averaging volume, as discussed in §2c. Since the size of the slip system averaging volume has been consistently kept the same throughout the present work, its effect will not be reflected in the fatigue life predictions. However, the reported value for $W_{\text{critical}}^{p}$ should not be considered as a benchmarked number for 718Plus. For more rigorous estimation of the $W_{\text{critical}}^{p}$, the following should be considered.

(a) Nonlinear finite elements should be used to avoid volumetric/shear locking and thus eliminating the necessity of the slip system-based averaging.
(b) The mesh near the grain boundary should be sufficiently refined to accurately capture the gradients associated with the mechanical field variables in that region.

The fatigue life was experimentally obtained using specimens with a gauge length and diameter of 12.7 mm and 5 mm, respectively, whereas the domain size for the CPFE simulations was restricted to cubes of 180 µm length owing to the higher computational cost. Since simulation domain size was kept identical for calibration of $W_{\text{critical}}^{p}$ as well as prediction of fatigue life, its effect is not seen in figures 8 and 9. In the present work, the uncertainty in $W_{\text{critical}}^{p}$ as a result of the size of the SEM is implicitly taken care of via the hyper-parameter through equation (4.2). In [48], it is shown that, if the number of grains within the SEM is around or greater than 150, convergence is achieved in the strength properties of the material (in terms of capturing the macroscopic yield, hardening behaviour, Bauschinger effect, etc.). In the present work, the consistent size of the SEMs is necessary to capture the effect of microstructure variability in the fatigue behaviour and thereby the fatigue performance, but each SEM is not designed to predict the statistical minimum fatigue life. The appropriate size of the microstructure instantiation, at the mesoscopic length scale, should be identified more rigorously for fatigue predictions at the length scale of a component, which is a topic for future work.

When the macroscopic stress–strain data are used to calibrate the crystal plasticity parameters, as in the present work, the resulting crystal plasticity parameters may not be unique, which would lead to additional uncertainty in the values of $w_{10}^{p}(x^{*})$ and $\Delta w_{10}^{p}(x^{*})$. Detailed uncertainty quantification of the crystal plasticity parameters and their influence on the associated fatigue metrics (e.g. the SPSED) have been performed by the authors, as reported in [62]. The results indicate that the uncertainty in the crystal plasticity parameters may propagate to approximately 25% variability in the values of $w_{10}^{p}(x^{*})$ and $\Delta w_{10}^{p}(x^{*})$ [62]. In the present context, it would imply that the calibrated distribution of $W_{\text{critical}}^{p}$ might result in a slightly higher standard deviation, which would not affect the mean of $W_{\text{critical}}^{p}$ and hence the predicted fatigue life.

As discussed in §1, the material stores a portion of its internal plastic work by forming dislocation structures and sub-structures, and the rest is primarily dissipated as heat energy. The stored portion of the internal plastic work is of interest in the context of fatigue failure. It is often argued that 5% of the total plastic work is stored in the material and the remaining 95% is lost as heat energy [50]. However, the number 95% is not universally accepted (see, for example, [85,86]); in some cases, it is found to be increasing with increasing plastic strain [85]. Hence, there exists a critical need to carry out rigorous studies to characterize the percentage of plastic work lost as heat energy and, in turn, the percentage of plastic work stored within the material as a function of applied strain and temperature. In the absence of a lack of agreement between the reported values of the partitioning coefficient in the literature, researchers often neglect the partitioning effect in their numerical calculations (see, for example, [7–31]). With this, while computing $W_{\text{critical}}^{p}$, we have not considered the partitioning of the plastic work. The entire plastic work has been assumed to be stored by the material. In the present context, such an assumption only affects the calibrated value of the $W_{\text{critical}}^{p}$, not the predicted fatigue lives. In other words, a more realistic calibration of $W_{\text{critical}}^{p}$ would require incorporation of a scalar constant in equations (3.1)–(4.1) to incorporate the partitioning aspect, which would change only the calibrated numerical value of $W_{\text{critical}}^{p}$ but not the subsequent fatigue life prediction.

One can observe from figure 9 that the predicted life at lower strain amplitude, especially at $R_{\varepsilon }=0.05$, spans from approximately 10^5^ cycles to approximately 10^6^ cycles. Therefore, if experimental data at these strain amplitudes are used to calibrate $W_{\text{critical}}^{p}$, the resulting posterior distributions will involve a higher level of uncertainty, characterized by a higher standard deviation in $W_{\text{critical}}^{p}$ and higher mean and standard deviation for the hyper-parameter *s*. In other words, just like the model discrepancy δ(β) and experimental uncertainty e(β) terms in equation (4.2), the hyper-parameter *s* is also dependent on the loading conditions defined by β. Since the life predictions from different SEMs involve least scatter at the 1.2% applied strain range, the experimental data corresponding to the same loading regime are used in the present work to calibrate $W_{\text{critical}}^{p}$. If the entire experimental and simulation datasets for $R_{\varepsilon }=-1$ are used for the calibration,^2^ the resulting mean and standard deviation of $W_{\text{critical}}^{p}$ are found to be approximately 4% lower and approximately 4 times higher, respectively, whereas the order of magnitude of the hyper-parameter is found to be approximately 100 times higher than the values reported in table 3. These observations further support the foregoing discussion.

**Figure 10.**
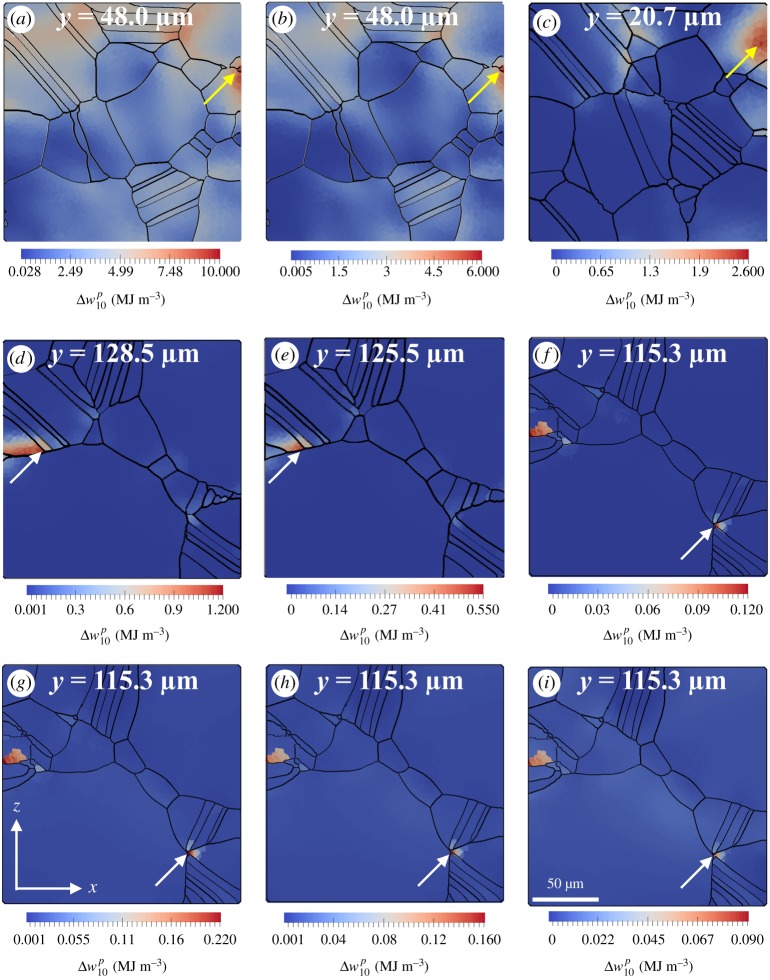
The location of maximum $\Delta w_{10}^{p}$ within SEM 1 at (*a*) $\Delta \varepsilon =1.2\%,\;R_{\varepsilon }=-1$, (*b*) $\Delta \varepsilon =0.95\%,\;R_{\varepsilon }=-1$, (*c*) $\Delta \varepsilon =0.75\%,\;R_{\varepsilon }=-1$, (*d*) $\Delta \varepsilon =0.675\%,\;R_{\varepsilon }=-1$, (*e*) $\Delta \varepsilon =0.625\%,\;R_{\varepsilon }=-1$, (*f*) $\Delta \varepsilon =0.5\%,\;R_{\varepsilon }=-1$, (*g*) $\Delta \varepsilon =0.5\%,\;R_{\varepsilon }=0.05$, (*h*) $\Delta \varepsilon =0.43\%,\;R_{\varepsilon }=0.05$, and (*i*) $\Delta \varepsilon =0.36\%,\;R_{\varepsilon }=0.05$. The yellow arrows (arrows in the top row of images) indicate the critical location near or at the free surface and the white arrows (arrows in the middle and bottom rows of images) indicate hotspots at the twin boundaries. Each colour map is a cross-sectional view of the SEM 1 on the *x–z* plane, normal to the loading direction *y*. (Online version in colour.)

It has already been mentioned in §5b that an increasing spread in the fatigue life predictions, from an identical set of SEMs, is observed with a decreasing applied strain. Such progressive uncertainty in predictions, from the same set of SEMs, with decreasing applied strain is certainly a manifestation of the change in $x^{*}$, i.e. failure location, with applied loading situation. To confirm, $x^{*}$ is tracked within SEM 1 with the change in applied loading. A shift of the failure location from the free surface to an interior twin boundary is observed with the decrease in applied strain (figure 10). At Δ*ε *= 1.2%, 0.95% and 0.675%, the failure location is observed near or at the free surface (figure 10*a–c*). Subsequently, at lower Δ*ε*, the failure location is identified at interior twin boundaries (figure 10*d–i*). The ability of the microstructure-sensitive plastic strain energy density criterion to capture (i) the shift in failure location, often seen in real experiments, and (ii) real fatigue life data with reasonable accuracy across various loading regimes certainly demands attention of the industry as well as academia to reduce the extensive time and cost associated with experimental fatigue life characterization.

It is well known that properties associated with the mechanical behaviour of materials are inherently microstructure dependent. In the present work, we have hypothesized the critical plastic strain energy density as a material property and have characterized its statistical distribution. While predicting fatigue lives, we have used the mean value, which is applicable for the current microstructure. By altering the statistical descriptors of the microstructure, a different mean value of the critical plastic strain energy density is expected. However, such dissimilarity has to be verified in future work. Further, for a given statistical description of the microstructure, the applicability of a single critical plastic strain energy density is also required to be tested and verified in (i) the presence of defects, such as pores and inclusions, and (ii) other loading conditions, including the role of environment, various operating temperatures and multi-axial applied loading.

## 7. Conclusion

A microstructure-sensitive critical plastic strain energy density is postulated to be the driving mechanism of fatigue crack initiation and is applicable to predict failure across several loading regimes. The model is inherently physics based, and requires only one parameter, namely the critical plastic strain energy density. This critical plastic strain energy density is calibrated using experimental fatigue life data under fully reversed type loading at 1.2% applied strain range via the Bayesian inference method. Subsequently, the calibrated critical energy value is used to predict fatigue lives at eight strain ranges including strain ratios −1 and 0.05. The key findings are summarized below.

— Predicted lives from nine statistically equivalent microstructures for each applied strain range are found to follow a lognormal distribution which is consistent with observations in traditional fatigue experiments.
— A good agreement is observed between the experimental fatigue life and the lognormal mean of the predictions at eight strain ranges including strain ratios −1 and 0.05.
— For a given strain ratio, increasing spread in the predictions from the identical set of statistically equivalent microstructures is observed with decreasing applied strain amplitude.

## Footnotes

1 While identifying **x***, an element is ignored if its centroid lies within 20 µm from the surfaces where boundary conditions are specified.

2 While using experimental fatigue life data over multiple strain ranges, spanning several orders of magnitudes of life, to calibrate the critical plastic strain energy density, the error in prediction should be scaled while estimating the likelihood function via equations (4.4) and (4.5) to eliminate the effect of varying order of magnitudes in error at different applied strains. Under such a situation, the scaled square of the error might be computed as ${\left((N_{\exp}-N_{\text{predict}})/10^{\text{order}}(N^{\text{expt}})\right)}^{2},$ where the order (*x*) function returns the order of magnitude of the number *x*, and is used in equation (4.5) to calculate the sum of square of errors.

## Data accessibility

The fatigue data required to reproduce the findings of this work (in terms of both model predictions and experimental results) are available to download from: http://dx.doi.org/10.17632/5w4ttmwb4v.1.
